# Standard diet and animal source influence hippocampal spatial reference learning and memory in congenic C57BL/6J mice

**DOI:** 10.21203/rs.3.rs-4582616/v1

**Published:** 2024-07-16

**Authors:** Damyan W. Hart, Mathew A. Sherman, Minwoo Kim, Ross Pelzel, Jennifer L. Brown, Sylvain E. Lesné

**Affiliations:** University of Minnesota; University of Minnesota; University of Minnesota; University of Minnesota; University of Minnesota; University of Minnesota

**Keywords:** hippocampus, spatial memory, working memory, microbiome, standard diet, birth location

## Abstract

**Background:**

Assessing learning and memory has become critical to evaluate brain function in health, aging or neurological disease. The hippocampus is crucially involved in these processes as illustrated by H.M.’s remarkable case and by the well-established early symptoms of Alzheimer’s disease. Numerous studies have reported the impact of gut microbiota on hippocampal structure and function using pro-, pre- and antibiotics, diet manipulations, germ-free conditions or fecal transfer. However, most diet manipulations have relied on Western diet paradigms (high fat, high energy, high carbohydrates). Here, we compared the impact of two standard diets, 5K52 and 2918 (6% fat, 18% protein, 3.1kcal/g), and how they influenced hippocampal learning and memory in adult 6-month-old congenic C57BL/6J mice from two sources.

**Results:**

Using a hippocampal-dependent task, we found that 5K52-fed mice performed consistently better than 2918-fed animals in the Barnes circular maze. These behavioral differences were accompanied with marked changes in microbiota, which correlated with spatial memory retention performance. We next tested whether 2918-induced alterations in behavior and microbiome could be rescued by 5K52 diet for 3 months. Changing the 2918 diet to 5K52 diet mid-life improved spatial learning and memory in mice. Shotgun sequencing and principal component analyses revealed significant differences at both phylum and species levels. Multivariate analyses identified *Akkermansia muciniphila* or *Bacteroidales bacterium M11* and *Faecalibaculum rodentium* as the strongest correlates to spatial memory retention in mice depending on the animal source. In both settings, the observed behavioral differences only affected hippocampal-dependent performance as mice fed with either diet did similarly well on the non-spatial variant of the Y-maze.

**Conclusions:**

In summary, these findings demonstrate the diverging effects of seemingly equivalent standard diets on hippocampal memory. Based on these results, we strongly recommend the mandatory inclusion of the diet and source of animals used in rodent behavioral studies.

## Introduction

In the past decade, accumulating evidence has supported a link between gut microbiome and learning and memory in the brain, most notably in the hippocampus^1,2^. This relationship is particularly intriguing in the context of aging and dementia, including Alzheimer’s disease related dementias, in which alterations of the microbiome and dysbiosis have been documented^2–6^.

A common thread within the field focused on the impact of Western diet (WD), enriched in saturated fat and added sugars, as likely confounding factor by promoting systemic and brain inflammation^7,8^. In both rodents and humans, WD consumption negatively impacts cognitive function, most notably hippocampal-dependent learning and memory processes^8–11^. In experimental studies using rodents, standard diet (SD) containing less than 20% calories from fat is classically compared with high fat diet (HFD) containing 35–60% calories from fat to evaluate the impact of metabolic changes on microbiome and memory function^8,12,13^. For example, decreases in the phylum *Bacteroidetes* and increases in *Firmicutes* and *Proteobacteria* linked to cognitive deficits result from HFD consumption^14,15^. Similarly, poorer cognitive flexibility was reported in C57BL/6J mice when fed with either HFD or high-sucrose diet, and microbiome analysis revealed strong correlations between cognitive function, reduced abundance of *Bacteroidetes* (*Bacteroidales* order) and elevated abundance of *Firmicutes* (*Clostridiales* order)^16^. To demonstrate a causal effect of the microbiome on neurocognitive behavior, Bruce-Keller and coworkers performed fecal/cecal transplantations from WD-fed mice to young SD-fed C57BL/6J mice^13^. The abundance of *Verrucomicrobia* species *Akkermansia muciniphila*, previously identified for its beneficial impact on metabolic, inflammatory, neuronal and cognitive functions^17–21^, was markedly lowered by ~ 5-fold in the WD mouse group. Importantly, hippocampal-dependent deficits can be observed within very short periods, e.g. 3–9 days, of WD consumption without altering animal body weight^22,23^. Together these data indicate that WD can perturb cognitive performance and hippocampal function in particular, independently of overt metabolic deficits.

While most diet manipulations have relied on comparing WD to SD paradigms to study their impact on the gut microbiome-brain axis, it is vastly assumed that all SD are equivalent and do not affect cognitive function. To fill this knowledge gap, we compared the impact of two standard diets, 5K52 and 2918 with seemingly equivalent compositions (6% fat, 18% protein, 3.1kcal/g), and how they influenced hippocampal learning and memory in two independent cohorts of adult 6-month-old congenic C57BL/6J mice. We selected these SD because 5K52 is routinely used at The Jackson Laboratory and because 2918 is routinely used in specific pathogen-free facilities at our institution. Briefly, we found in our first cohort that mice initially fed with 5K52 but transitioned to 2918 for 1 or 3 months displayed a deficit in spatial reference memory compared to mice uniquely consuming 5K52. These behavioral differences were accompanied with notable gut microbiome alterations, including species which correlated with memory retention. In an independent cohort, we tested the ability of rescuing the cognitive and microbiomal phenotypes detected in 2918-fed mice by transitioning animals initially fed with 2918 to 5K52 for 3 months. We observed a partial rescue conferred by this transition resulting in an amelioration of hippocampal-dependent performance and microbiome composition. To our knowledge, these results are the first to document the differential impact of SD on hippocampal memory and gut microbiome.

## Materials and Methods

### Animals.

Congenic C57BL/6J mice were either newly purchased from The Jackson Laboratory (Strain #: 000664) or generated from congenic C57BL/6J colonies at the University of Minnesota established from C57BL/6J mice previously purchased from The Jackson Laboratory (Strain #: 000664). Accordingly, two cohorts of C57BL/6J mice, termed JAX or UMN, were used based on their birth location and kept segregated. Only male animals were used for behavioral testing and microbiome sequencing.

At weaning, pups were housed in same-sex cohorts (2 to 4 animals per cage). C57BL/6J mice were housed in a mouse barrier facility under specified pathogen-free (SPF) conditions until they were euthanized at 6–6.5 months of age post-behavioral testing.

C57BL/6J mice were either given 5K52 pellets (5K52 - LabDiet^®^ JL Rat and Mouse/Auto 6F, LabDiet^®^) or 2918 pellets (2918 Teklad Irradiated Global 18% Protein Rodent Diet) *ad libitum*. For the 5K52 (3M) −> 2918(1M) and the 5K52 (3M) −> 2918 (3M) groups, mice were fed with 5K52 *ad libitum* until 3 months of age then given 2918 for 1- or 3-months *ad libitum*. For the 2918 (3M) −> 5K52 (3M) group, mice were fed with 2918 *ad libitum* until 3 months of age then given 5K52 for 3 months *ad libitum*. A summary of the key differences between these standard diets can be found in Table 1.

All groups of animals were housed under the same SPF conditions: same type of cage (Cat. # 75Jag (PC75JHT); Allentown, LLC; Allentown, NJ), bedding materials (Cat. # 7092; Inotiv^®^ Teklad Irradiated Corncob Bedding 1/8”; Inotiv; West Lafayette, IN), and environmental enrichment (Enviro-dri^®^; Shepherd, Inc; Watertown, TN) in the same room in the vivarium (1–506 WMBB; University of Minnesota, Minneapolis, MN).

All animal procedures and studies were reviewed and approved by the University of Minnesota Institutional Animal Care and Use Committee and Institutional Review Board, and the animals care was in accordance with institutional guidelines.

### Barnes circular maze.

The apparatus used was an elevated circular platform (0.91 m in diameter) with 20 holes (5 cm diameter) around the perimeter of the platform, one of which was connected to a dark escape recessed chamber (target box) (San Diego Instruments, USA). The maze was positioned in a room with large, simple visual cues attached on the surrounding walls. The protocol used here was published elsewhere (http://www.nature.com/protocolexchange/protocols/349). Briefly, mice were habituated to the training room prior to each training day for 30 minutes in their cages. In addition, on the first day mice were placed at the center of the maze in a bottomless opaque cylinder for 60 sec to familiarize the animals with the handling. Training sessions started 15 minutes later. Acquisition consisted of 4 trials per day for 4 days separated by a 15-minute inter-trial interval. Each mouse was positioned in the center of the maze in the opaque cylinder, which was gently lifted and removed to start the session. The mice were allowed 180 seconds to find the target box on the first trial; all trials were 3 minutes long. At the end of the first 3 minutes, if the mouse failed to find the recessed escape box, it was gently guided to the chamber and allowed to stay in the target box for 60 seconds. The location of the escape box (green circles on track plots) was kept constant with respect to the visual cues. An animal was considered to find the escape chamber when its head and shoulders were above the escape box. Memory retention was tested 24 hours after the last training session (Probe trial day 5). Briefly, the escape hole was blocked for the duration of the probe, and the time spent searching the target quadrant for the escape hole was measured for each mouse. The same parameters were collected during acquisition and retention phases using the ANY-maze software (Stoelting Co., USA).

### Y-maze task.

Short-term working memory was tested using an enclosed Y-maze. In this non-spatial variant of the task, mice freely explore all three arms of the maze, driven by their innate curiosity to visit previously unexplored areas (Lalonde, 2002). Testing was performed using a Y-shaped maze with three white-colored, opaque arms orientated at 120° angles from each other. Mice were introduced in the starting arm (C arm) and allowed to explore the maze over 5 mins (single trial). An entry occurs when all four limbs of the mouse are within an arm. An alternation is defined as consecutive entries into all three arms. Animal tracking, the number of arm entries and alternations were recorded using ANY-maze software (Stoelting Co., USA) and these parameters were used to measure of spontaneous alternation in the Y-maze. A high percent-age alternation was thus defined by a high proportion of entries into consecutive arms (e.g., A->B->C arms). By contrast, a low spontaneous alternation percentage was defined by a higher proportion of repeated entries into the same arm, indicative of poor working memory.

Two male experimenters, MAS and DWH, ran the behavioral tasks summarized in Supplementary Figs. 2 and 6 respectively.

### Shotgun whole-genome sequencing.

Two fecal pellets were collected per mouse and samples were sent to TransnetYX (Cordova, TN) for DNA extraction, library preparation, and shallow shotgun whole-genome sequencing. Sequencing data were uploaded automatically onto the One Codex microbiome analysis platform, which was used to extract the fecal microbiome relative abundance data. The OneCodex raw data are available in Supplementary Table 1.

### Statistical Analyses.

All data were expressed as mean values ± SEMs. Analyses were performed using JMP Pro 17 (SAS Institute, USA). When variables were non-normally distributed, nonparametric statistics were used (Spearman correlation coefficients, Kruskal-Wallis non-parametric analysis of variance followed by *posthoc* Mann-Whitney U or Steel-Dwass tests for three group comparisons). When variables were normally distributed, the following parametric statistics were used (Pearson’s correlation, one/two/three-way ANOVA or RM-ANOVA followed by Bonferroni-corrected two-group *posthoc* Student *t* tests or Tukey HSD test for three group comparisons). Hierarchical clustering and Benjamini-Hochberg False Discovery Rate (FDR) statistics were applied for multiple testing analyses of microbiome datasets. Robust FDR was used to minimize outlier effects. The null hypothesis was rejected at the *P* < 0.05 level. Statistical tests and outcomes are compiled in Supplementary Table 2.

## Results

### Standard diets influence spatial learning and memory performance

**Figure 1:**
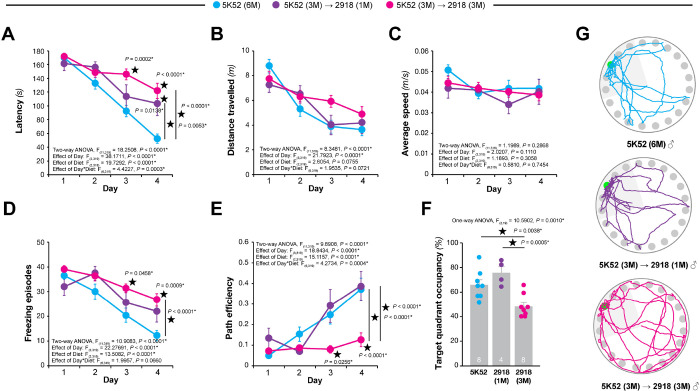
Behavioral effects of standard diets 5K52 and 2918 in young adult male C57BL/6J mice. Male C57BL/6J animals were tested in the Barnes circular maze (BCM) at four to six months of age. (**A, B**) Spatial learning of the BCM task was reflected by reductions in escape latency (*A*) and distance traveled (*B*). (**C, D**) Measurements of average animal speed (*C*) and freezing episode numbers (*D*) in the BCM task were used to assess phenotypic changes other than spatial learning. (**E**) Quantitative assessment of animal trajectories during the learning phase using path efficiency. (**F, G**) Spatial memory retention in the BCM task was determined by target quadrant occupancy (*F*) and by assessment of path traces (*G*). Data represent mean ± SEM; 〿*P* < 0.05, n = 4–8 mice/group.

To determine the influence of standard diets on learning and memory, adult three-month-old male C57BL/6J JAX mice were either fed with 5K52 for 3 months or with 2918 for 1- or 3-months (**Suppl. Figure 1**) and subjected to the Barnes circular maze (Fig. 1) and the Y-maze (**Suppl. Figure 2**). Contrary to mice fed with 5K52 for 6 months, 5K52 (3M) −> 2918 (3M) mice displayed a prolonged latency to find the escape hole (Fig. 1A). Mice fed for 1 month with 2918 also displayed longer latencies albeit between 5K52 and 5K52 (3M) −> 2918 (3M) groups (Fig. 1A). Distance travelled and average speed did not differ across these groups arguing against differences in motor function (Fig. 1B–C). Freezing episodes were more numerous in 5K52 (3M) −> 2918 (3M) mice compared to 5K52 animals suggesting a potential differential stress response (Fig. 1D). Using path efficiency as an index of spatial learning (the higher the number, the more targeted the path is), both 5K52 and 5K52 (3M) −> 2918 (1M) groups performed similarly. However, 5K52 (3M) −> 2918 (3M) mice displayed much lower path efficiencies (Fig. 1E). Integrating latency and path efficiency parameters, we conclude that 5K52 (3M) −> 2918 (3M) animals present with spatial reference learning deficits compared to 5K52-fed mice. The following day, spatial memory retention was assessed in a probe trial and target quadrant occupancy (Fig. 1F) and corresponding track plots (Fig. 1G) revealed that 5K52 (3M) −> 2918 (3M) mice were poorer performers compared to 5K52 and 5K52 (3M) −> 2918 (1M) groups.

Short-term working memory was also evaluated in 5K52 and 5K52 (3M) −> 2918 (3M) groups using a non-spatial Y-maze (**Suppl. Figure 2**). Spontaneous alternation, total arm entries and total alternations were similar across groups as well as distance traveled, average speed and total time mobile indicating that working memory was not impaired.

These results thus demonstrated that 5K52 (3M) −> 2918 (3M) mice display spatial reference memory learning and retention deficits compared to 5K52 mice.

### Standard diet change alters the gut microbiome

**Figure 2:**
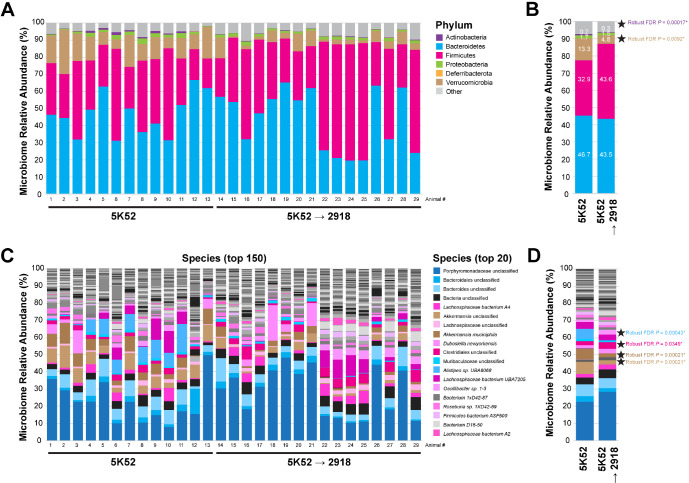
Gut microbiome composition at the phylum and species levels in C57BL/6J mice from the JAX cohort. Mice from the JAX cohort were fed with 5K52 for 6 months (“5K52”) or with 5K52 for 3 months and then transitioned to 2918 for the next 3 months (“5K52 (3M) → 2918 (3M)”). (**A**) Stacked histogram plot depicting the relative abundance top 6 phyla (*Bacteroidetes, Firmicutes, Verrucomicrobia, Proteobacteria, Actinobacteria* and *Deferribacterota*) in 29 mice. The remaining phyla are included in the “Other” category. (**B**) Stacked histogram depicting the averaged relative abundance of the most represented phyla in 5K52 and 5K52 → 2918 groups. (**C**) Stacked histogram plot depicting the relative abundance top 150 species in 29 mice. The top 20 most represented species are colored as indicated in the legend. (**D**) Stacked histogram depicting the averaged relative abundance of the most represented species in 5K52 and 5K52 → 2918 groups. Data represent means (n_5K52_ = 13 mice, n_5K52 → 2918_ = 16 mice). When present, numbers in white font correspond to the mean group values for a given taxon. 〿Robust FDR *P* values < 0.01.

To determine potential effects of the change in the seemingly equivalent standard diets on the gut microbiome composition, fecal samples were subjected to whole-genome sequencing. As behavioral differences were primarily seen between the 5K52 and the 5K52 (3M) −> 2918 (3M) mice, our microbiome analyses focused on these groups. Alpha diversity metrics (Shannon, Simpson and observed taxa) revealed no differences between groups (**Suppl. Figure 3**). However, when comparing 5K52 (3M) −> 2918 (3M) to 5K52 mice at the phylum level, we found that the relative abundance of *Verrucomicrobia* (−8.472%) and *Actinobacteria* (−0.473%) were altered (Fig. 2A–B).

No overt changes in *Bacteroidetes, Firmicutes, Proteobacteria* and *Deferribacterota* were observed, phyla previously linked to changes in cognitive performance^14–16^. At the species level, the evaluation of the fecal microbiome composition of each group identified differences in 7 classified and 3 unclassified species (Fig. 2C–D and Table 2). It is worth noting that 45.5% (5/11) displayed reduced abundance while 54.5% (6/11) showed a greater abundance in 5K52 (3M) −> 2918 (3M) compared to 5K52 mice. Amongst them, the relative abundance of *Alistipes* sp. UBA6068 (−6.461%) and *Akkermansia muciniphila* (−4.602%) was reduced the most and *Lachnospiraceae bacterium UBA7188* (+ 0.331%) and bacterium 1XD21–70 (+ 0.253%) were increased the most across groups.

Reducing the dimensionality of the phylum and species dataset using principal component analysis (PCA) segregated 5K52 and 5K52 (3M) −> 2918 (3M) groups (**Suppl. Figures 4,5**). At the phylum level, the principal component 1 (PC1) which accounted for 21% of the variance distinguished groups by diet but not PC2. By contrast, PC1 and PC4 both differed across groups (**Suppl. Figure 4A,B**), which was further supported by the partial contribution plots of PC1, PC2 and PC4 (**Suppl. Figure 4C,D**). At the species level, PC1 accounted for 43% of the variance readily discriminated the diet groups but PC2 did not (**Suppl. Figure 5A**). Combining PC1 and PC5 allowed the segregation of 5K52 and 5K52 (3M) −> 2918 (3M) groups (**Suppl. Figure 5B**). Partial contribution plots revealed that *Akkermansia muciniphila* and unclassified Akkermansia were more represented in PC1 and in PC5 than in PC2 (**Suppl. Figure 5D**) possibly hinting that its relative abundance might be critical when considering differences across diet groups in the JAX cohort.

These findings indicated that gut microbiome profiling discriminates 5K52 and 5K52 (3M) −> 2918 (3M) mouse groups and that a simple standard diet change can result in substantial modifications of the microbiota composition.

### Spatial memory retention positively correlates with Akkermansia muciniphila abundance

**Figure 3:**
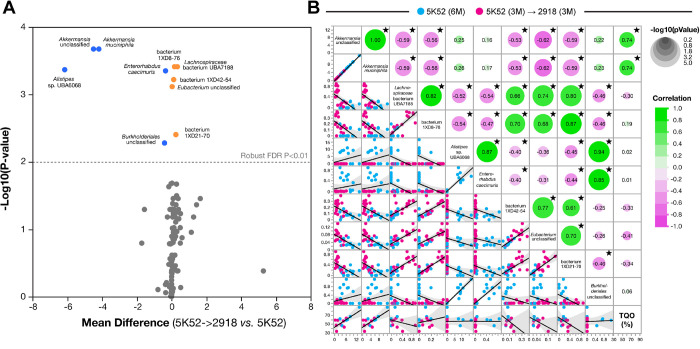
Unclassified *Akkermansia* and *Akkermansia muciniphila* are associated with spatial memory retention in C57BL/6J mice from the JAX cohort. (**A**) Volcano plot depicting the differentially abundant species across diet groups 5K52 → 2918 and 5K52. Species reduced across groups are displayed in blue, while species increased across groups are colored in orange. The robust FDR P < 0.01 cutoff is indicated on the panel by a dashed grey line. All species below that threshold are colored grey. (**B**) Scatterplot matrix illustrating the associations between all differentially-abundant species (Robust FDR P-values < 0.01) and target quadrant occupancy in the BCM [TQO(%)]. Blue dots correspond to 5K52 mice while pink dots indicate mice from the 5K52 −> 2918 group. Fit lines and 0.95 confidence intervals (grey shade) are shown in the left bottom half while significance circles and correlation values are shown in the right top corner. 〿*P* < 0.05, n = 29 mice.

To determine putative relationships between the relative abundance of gut microbiome species and hippocampal-dependent spatial reference memory, we performed a multivariate analysis between the differentially abundant species (Fig. 3A) and target quadrant occupancy (TQO) (Fig. 3B). Expectedly, the relative abundance of *Akkermansia* species (unclassified *Akkermansia* and *Akkermansia muciniphila*) and unclassified bacteria species (bacterium 1XD8–76, bacterium 1X42–54, bacterium 1XD21–70) positively correlated with each other while that of *Enterorhabdus caecimuris*, an *Actinobacteria*, was unrelated. Out of these 10 species, only unclassified *Akkermansia* and *Akkermansia muciniphila* were correlated to TQO in a statistically significant manner (*P* = 0.0025 and *P* = 0.0026 respectively) (Fig. 3B).

These results thus associate a higher abundance of *Akkermansia* species with better spatial memory retention in young adult JAX mice fed with 5K52 for 6 months compared to mice fed with 5K52 for 3months and 2918 for the next 3 months.

### Partial rescue of spatial memory deficits in mice transitioned from 2918 to 5K52

**Figure 4:**
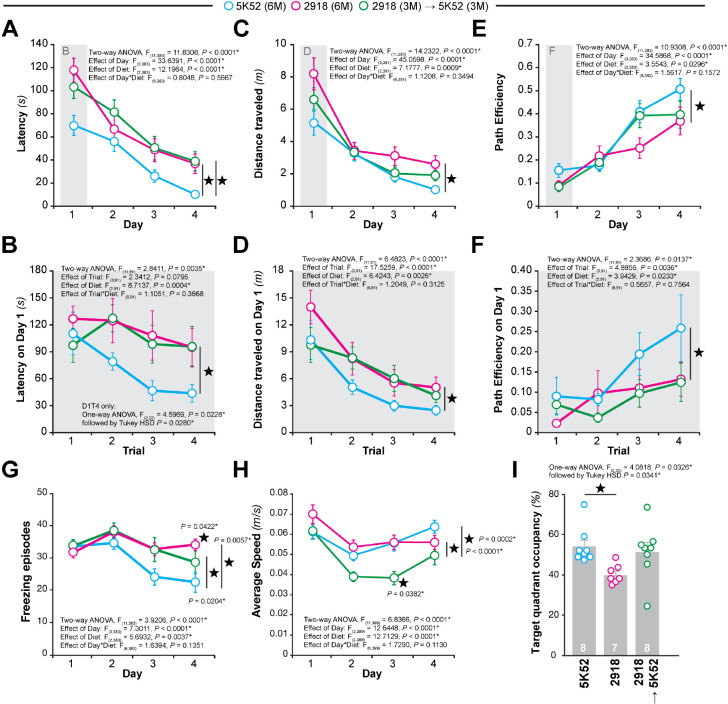
Partial rescue of behavioral deficits in young adult male C57BL/6J mice transitioning from 2918 to 5K52 diet. Male C57BL/6J animals from the UMN cohort were tested in the Barnes circular maze (BCM) at six months of age. Three groups were compared: animals fed with 5K52 for 6 months (5K52), animals fed with 2918 for 6 months (2918) and mice fed with 2918 for 3 months and 3 months with 5K52 (2918 → 5K52). (**A-D**) Spatial learning of the BCM task was reflected by reductions in escape latency (*A,B*) and distance traveled (*C,D*). Data are presented over days (*A, C*) or trials (*B, D*). (**E, F**) Quantitative assessment of animal trajectories during the learning phase using path efficiency. (**G, H**) Measurements of freezing episode numbers (*G*) and average animal speed (*H*) in the BCM task were used to assess phenotypic changes other than spatial learning. (**I**) Spatial memory retention in the BCM task was determined by target quadrant occupancy. Data represent mean ± SEM; 〿*P* < 0.05, n = 7–8 mice/group.

Because feeding C57BL/6J mice with 2918 after 3 months of age caused a decline in spatial memory performance, we sought to determine whether feeding 5K52 in mice raised on 2918 for 3 months could rescue the potential observed memory deficits. To this end, a new experimental cohort of C57BL/6J mice, coined UMN cohort, was either fed with 5K52 for 6 months, 2918 for 6 months or 2918 for 3months followed by 5K52 for 3 months [2918 (3M) −> 5K52 (3M)] (**Suppl. Figure 1**). All three groups fed with these standard diets were subjected to the Barnes circular maze (Fig. 4) and Y-maze (**Suppl. Figure 6**) tasks to assess spatial reference memory and short-term working memory. In the Barnes maze, the 5K52 group displayed the shortest latency to find the escape hole across all four days of spatial memory acquisition compared to 2918 and to 2918 (3M) −> 5K52 (3M) groups (Fig. 4A).

Because the average latencies of the 5K52 group already differed on the first day of training, we postulated that 5K52-fed mice might display an accelerated learning compared to 2918 and to 2918 (3M) −> 5K52 (3M) groups. Examining trial performance on day 1 confirmed that all groups started with equivalent average latencies on trial 1 (Fig. 4B). However, the 5K52 group reached ~ 45 second latencies in trials 3–4 of Day 1 while mice from the other two groups hovered above 90 seconds, indicating that 5K52-fed mice learned this spatial task much faster than 2918 groups. Like latency, distance traveled can also reflect spatial memory acquisition. Once more, a marked effect of diet was observed whereby 5K52 covered shorter distances to complete the task whereas mice fed 2918 for 6 months ran the longest (Fig. 4C). Interestingly, the 2918 (3M) −> 5K52 (3M) mice were undistinguishable from 5K52 mice across all four days or across trials on day 1 (Fig. 4C,D). We next examined path efficiency across diet groups (Fig. 4E,F). Consistent with shorter latencies and distance traveled, mice fed with 5K52 displayed the highest path efficiencies while mice with 2918 for 6 months were the worst in this spatial measure of learning. Again, 5K52 mice relied on better path efficiencies already on day 1 to find the target hole as further demonstrated by trial-by-trial performance on day 1 (Fig. 4F). Together, latency, distance traveled, and path efficiency measures demonstrate that mice fed 5K52 for 6 months learned the task best, mice fed 2918 for 6 months were the worst learners and 2918 (3M) −> 5K52 (3M) mice presented an intermediate phenotype.

Because feeding 2918 for 3 months in JAX mice elevated freezing possibly indicating a differential stress response caused by the diet change, we also monitored freezing episodes in the UMN cohort (Fig. 4G). In both groups of mice fed with 2918, freezing events were more numerous compared to 5K52 animals reproducing the observed change in behavior detected in the JAX cohort (Fig. 1D). Analysis of average speed did not track with freezing but instead revealed that 2918 (3M) −> 5K52 (3M) mice were noticeably slower compared to both 5K52-fed or 2918-fed mice (Fig. 4H).

Spatial memory retention was tested in the probe administered on day 5 (Fig. 4I). In line with the learning trajectories described above, 5K52 mice had the highest TQO (52.7 ± 3.1%) whereas 2918 mice had the lowest (39.6 ± 3.5%). Mice which transitioned from 2918 to 5K52 after 3 months displayed TQO reminiscent of the 5K52 group performance (50.6 ± 3.3%), suggesting that the standard diet change to 5K52 rescued the spatial memory retention deficits caused by the 2918 diet.

Short-term working memory was also evaluated in 5K52, 2918 and 2918 (3M) −> 5K52 (3M) groups using a non-spatial Y-maze (**Suppl. Figure 6**). Spontaneous alternation, total arm entries and total alternations were similar across groups. However, we observed notable increases in distance traveled, average speed and total time mobile in the mice fed 2918 since birth (**Suppl. Figure 6D-E**), reflecting a hyperactive phenotype which possibly resulting from an enhanced stress response in the task.

Altogether, these results indicated that C57BL/6J UMN mice fed with 2918 for 6 months display spatial reference memory learning and retention deficits compared to 5K52 mice. In addition, these 2918-induced deficit could be partially rescued by switching diet for 3 months.

### The behavioral rescue resulting from transitioning diet from 2918 to 5K52 alters the gut microbiome composition

**Figure 5:**
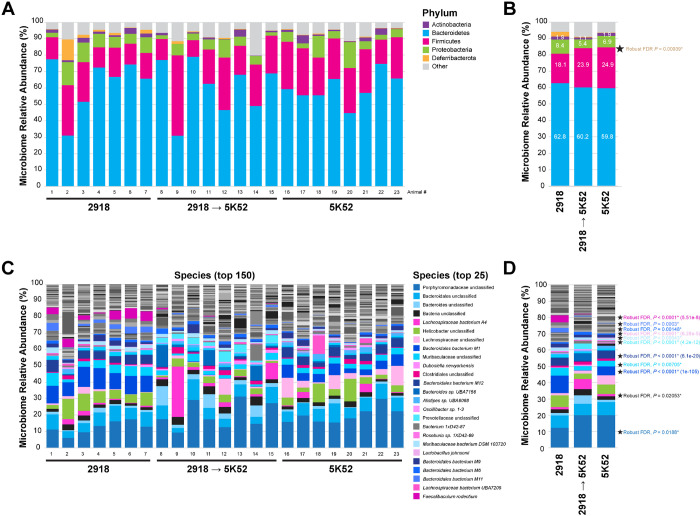
Gut microbiome composition at the phylum and species levels in C57BL/6J mice from the UMN cohort. Mice from the UMN cohort were fed with 5K52 for 6 months (5K52), with 2918 for 6 months (2918) or fed with 2918 for 3 months and 3 months with 5K52 (2918 → 5K52). (**A**) Stacked histogram plot depicting the relative abundance top 5 phyla (*Bacteroidetes, Firmicutes, Proteobacteria, Actinobacteria* and *Deferribacterota*) in 23 mice. The remaining phyla are included in the “Other” category. (**B**) Stacked histogram depicting the averaged relative abundance of the most represented phyla in 5K52, 2918 and 2918 → 5K52 groups. Note that *Verrucomicrobia* are not visible due to their extremely low abundance (< 0.002%) in all three groups. (**C**) Stacked histogram plot depicting the relative abundance top 150 species in all 23 mice. The top 25 most represented species are colored as indicated in the legend. (**D**) Stacked histogram depicting the averaged relative abundance of the most represented species in 5K52, 2918 and 2918 → 5K52 groups. Data represent means (n5_K52_ = 7 mice, n_2918_ = 8 mice, n_2918 → 5K52_ = 8 mice). When present, numbers in white font correspond to the mean group values for a given taxon. Robust FDR statistics were applied, and robust FDR *P* values are listed.

To determine potential effects of the transitioning the 2918 diet to the 5K52 standard diet change on the gut microbiome composition in the UMN cohort, fecal samples were subjected to whole genome sequencing as reported for the JAX cohort. Alpha diversity metrics (Shannon, Simpson and observed taxa) revealed no differences between groups (**Suppl. Figure 7**). At the phylum level, we found no overt changes in the relative abundance of *Bacteroidetes, Firmicutes, Proteobacteria, Actinobacteria* and *Deferribacterota*, which were the top 5 most abundant phyla, whereas that of *Verrucomicrobia* differed across groups (*P* = 0.00039) (Fig. 5A,B).

However, compared to the JAX cohort, the representation of *Verrucomicrobia* was nearly non-existent, below 0.005% in all three groups (**Supplementary Fig. 8**). At the species level, the evaluation of the fecal microbiome composition of each group identified differences in 35 classified and 9 unclassified species (Fig. 5C–D and Table 3).

Amongst those, the most abundant species included unclassified *Porphyromonadaceae*, which accounted for ~ 20% in both 5K52 and 2918 (3M) −> 5K52 (3M) groups (19.95 ± 2.15 and 19.94 ± 2.15 respectively) but only ~ 12% (12.35 ± 2.29) in 2918 mice. By contrast, *Bacteroidales* bacterium M1 abundance was highest in the 2918 group (10.80 ± 0.51%) compared to both 5K52 and 2918 (3M) −> 5K52 (3M) groups (5.41 ± 0.47 and 0.08 ± 0.47 respectively).

PCA further confirmed the segregation of the three diet groups at both phylum (**Suppl. Figure 9**) and species level (**Suppl. Figure 10**). In the former, PC1 and PC2 differentiated the 2918 (3M) −> 5K52 (3M) group from the animal groups fed with either 5K52 or 2918 but failed to separate these two groups (**Suppl. Figure 9A**). By contrast, combining PC2 and PC5 allowed to differentiate all three groups (**Suppl. Figure 9B**), possibly due to the notable representation of Firmicutes in partial contribution plots of PC1 but not PC2 and PC5, which were dominated by *Deferribacterota*, *Bacteroides* and *Proteobacteria* (**Suppl. Figure 9D**). At the species level, PC1 accounting for nearly 30% of the variance failed to segregate all groups while PC2 discriminated the 2918 (3M) −> 5K52 (3M) group well (**Suppl. Figure 10A**). Combining PC2 and PC3 allowed a clear segregation of all three diet with minimal overlap across clusters (**Suppl. Figure 5B**). Partial contribution plots revealed that *Bacteroidales* bacterium M2, uniquely detected in the 2918 (3M) −> 5K52 (3M) group, and *Bacteroidales* bacterium M6, elevated by about two fold in that same group, were more represented in PC2 while *Faecalibaculum rodentium*, unclassified *Porphyromonadaceae* and *Bacteroidales* bacterium M11 comprised notable representations in PC3 (**Suppl. Figure 5D**) possibly hinting that their relative abundance might be critical when considering differences across diet groups in the JAX cohort.

These findings indicated that gut microbiome profiling discriminates 5K52, 2918 and 2918 (3M) −> 5K52 (3M) mouse groups and that a simple standard diet change can result in substantial modifications of the microbiota composition.

### Bacteroidales bacterium M11, M13 and Faecalibaculum rodentium are the strongest correlates to spatial memory retention in the UMN mouse cohort

**Figure 6:**
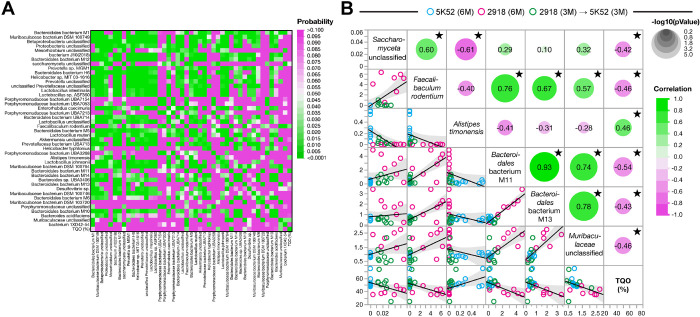
In quasi-absence of *Akkermansia*-like species, *Bacteroidales* bacterium M11, M13 and *Faecalibaculum rodentium* are the strongest correlates to spatial memory retention in the UMN mouse cohort. (**A**) Probability heatmap indicating the relationships between all differentially-abundant species (Robust FDR P-values < 0.01) in the 5K52, 2918 and 2918 → 5K52 groups from the UMN cohort and target quadrant occupancy in the BCM [TQO(%)]. (**B**) Scatterplot matrix illustrating the associations between the 6 species identified by the multivariable analysis and TQO(%). Blue dots correspond to 5K52-fed mice, pink dots indicate 2918-fed mice and green dots correspond to mice from the 2918 −> 5K52 group. Fit lines and 0.95 confidence intervals (grey shade) are shown in the left bottom half while significance circles and correlation values are shown in the right top corner. 〿*P* < 0.05, n = 23 mice.

To determine putative relationships between the relative abundance of gut microbiome species and hippocampal-dependent spatial reference memory in the UMN cohort, we performed a multivariate analysis between all 44 differentially abundant species and target quadrant occupancy (TQO) (Fig. 6).

The probability coefficient heatmap identified 6 species, namely *Bacteroidales* bacterium M11 and M13, *Faecalibaculum rodentium (F. rodentium), Alistipes timonensis (A. timonensis)* and unclassified *Muribaculaceae* and saccharomyceta (Fig. 6A). The scatterplot matrix for these variables was thus examined for their relationship with TQO, used as index of spatial memory performance, was confirmed (Fig. 6B). The abundance of species within the Bacteroidetes phylum were highly correlated to each other, except for *A. timonensis*. It is worth nothing that the abundance of *F. rodentium* (Firmicutes) was also strongly correlated to all Bacteroidetes apart from *A*. *timonensis*. Out of these 6 species, the abundance of the *A. timonensis* was positively correlated to TQO while the rest was negatively correlated with TQO (Fig. 6B).

Therefore, these results link the abundance of 6 species with better spatial memory retention in young adult UMN mice fed with 5K52 for 6 months and in mice fed with the “rescue” diet regimen, i.e., 3 months of 2918 feed following by 3 months of 5K52, compared to mice fed with 2918 for 6 months.

## Discussion

The accumulating evidence supporting the effects of the gut microbiome on brain function and specifically hippocampal-dependent learning and memory is well documented^2,3,8,17,24^. Numerous studies relying on probiotics, prebiotics, antibiotics, diet manipulations, germ-free conditions or fecal/cecal transfer have indeed reported the impact of the gut microbiota on hippocampal structure and function (see for review^2,8^). Most experimental diet manipulations have leveraged paradigms modeling WD versus SD consumption. While this approach is valid, it assumes that standard diets (SDs) are equivalent across providers and sources. Considering the well-established effect of microbiota composition on hippocampal structure and function, we hypothesized that seemingly equivalent SDs (6% fat, 18% protein, 3.1kcal/g), 5K52 and 2918, could influence hippocampus-dependent spatial learning and memory in young adult 6-month-old congenic C57BL/6J mice. We selected these SDs because 5K52 is routinely used at The Jackson Laboratory (JAX), a worldwide provider and pioneering organization for mouse modeling of human disease, and because 2918 is routinely used by Research Animal Resources in specific pathogen-free facilities at our institution, the University of Minnesota (UMN). Because microbiota composition depends on the birth environment in humans and mice^25–29^, our studies included two independent cohorts of congenic C57BL/6J male mice, one born at JAX and one born at UMN, which were maintained in SPF facilities. In the JAX cohort, we found that adult mice fed with 5K52 from birth to 3 months of age but transitioned to 2918 presented with spatial learning and retention memory deficits compared to mice continuously fed with 5K52. We also found that short-term working memory was intact across groups in JAX mice using a non-spatial variant of the YM, indicating that the spatial memory impairment was only observed in the BCM, a hippocampal-dependent task. Shotgun analysis identified alterations in the microbiome composition of 5K52->2918 mice, including increased abundance in *Firmicutes* and decreased abundances of *Verrucomicrobia* and Actinobacteria by over two-fold. The observed increase in *Firmicutes* and decrease in *Verrucomicrobia* are reminiscent of microbiome changes reported in WD-fed animals and these shifts have been associated with cognitive impairments^13–15^. Interestingly, amongst all species altered by the SD change in our cohort, the abundance of *Akkermansia muciniphila* was the only significant correlate with memory retention performance using target quadrant occupancy in JAX mice. The relationship of *A. muciniphila* with memory function has received recent interest in the neuroscience field due to its potential beneficial impact on various metabolic conditions and aging^30,31^. Specifically, the probiotic administration of *A. muciniphila* reversed cognitive impairment in WD-fed rats^18^ and in sleep-deprived C57BL/6N mice^32^. In male C57BL/6J mice, *A. muciniphila* was lowered by ~ 5-fold in WD-fed mice compared to mice consuming SD^13^. The beneficial effect of *A. muciniphila* on memory function in rodents were reported in three additional studies in which oral delivery of *A. muciniphila* prevented WD-induced memory deficits detected in a spatial variant of the YM^19^, BCM^20^, and novel objective recognition task and Morris water maze^18^. In experimental models of disease, oral gavage with *A. muciniphila* in the APP/PS1 mouse model of Alzheimer’s disease reduced soluble amyloid-beta concentrations in brain tissue and improved cognitive performance in the Y-maze of 9-month-old mice^33^. Thus, our findings in the JAX cohort are consistent with these independent studies, supporting a strong impact of *A. muciniphila* abundance on spatial reference memory performance.

Because JAX mice fed with 2918 for 3 months displayed cognitive deficits compared to mice which continuously consumed 5K52, we sought to test whether transitioning from 2918 to 5K52 would rescue the spatial memory impairments induced by 2918 in the independent UMN cohort of congenic C57BL/6J mice. Expectedly, 5K52-fed UMN mice performed best in the BCM task, 2918-fed UMN mice were the worst performers while mice switching SD in the 2918->5K52 group displayed a partial rescue of the 2918-induced deficits. Unlike in the JAX cohort, microbiome changes were only identified at the species level across SD groups. Amongst species altered by SD, *Bacteroidales bacterium M11, M13* and *F. rodentium* were the strongest correlates to spatial memory retention in the UMN mouse cohort. Notably, *A. muciniphila* was not associated with spatial memory retention in this second cohort.

*A. muciniphila* abundance comprises 1–4% of the human intestinal microbiota^21,34^ and *Verrucomicrobia Akkermansia* species share 80–95% homology with *A. muciniphila* in mammals, including mice^34^. In our JAX cohort, unclassified *Akkermansia* and *A. muciniphila* abundance accounted for 6.9% and 6.5% respectively in 5K52-fed mice, while feces from mice fed with 2918 contained 2.4% and 2.2%. By contrast, 5K52-fed mice in our UMN cohort showed much lower abundance of unclassified *Akkermansia* and *A. muciniphila*, 0.0019% and 0.0017% respectively. Given that both cohorts were fed with the same SD, the 3,700-fold difference in these *Verrucomicrobia* species across JAX and UMN cohorts illustrates the critical impact of birth location across cohorts. In addition, the reduction in *A. muciniphila* abundance in 2918-fed JAX mice compared to 5K52-fed JAX mice suggests either that 2918 chow inhibits or restricts in *A. muciniphila*’s representation in the gut or conversely that the 5K52 diet promotes *A. muciniphila* abundance. Considering that *A. muciniphila*’s presence was not detected in half of 5K52-fed UMN mice and when present, its relative abundance was below 0.0005% (**Suppl. Figure 10**), we interpret that the SD 5K52 alone is not sufficient to promote *A. muciniphila*’s intestinal presence in UMN mice. Unclassified *Akkermansia* species were detected across diet groups in the UMN cohort, but their abundance was again dwarfed by that observed in our independent JAX cohort. We therefore conclude that the relative abundance of the *Verrucomicrobia Akkermansia muciniphila* in C57BL/6J mice is principally dictated by its birth environment. This conclusion is further supported by a recent independent study which described the composition of the fecal microbiota of male and female C57BL/6J mice purchased from The Jackson Laboratory^35^. There, Roman and colleagues used 2-month-old mice born at The Jackson Laboratory for their study, fed their mice with 5053, a 4.5% fat, 20% protein, 3.1kcal/g diet from the same manufacturer as 5K52 (LabDiet), and used the same One Codex microbiome analysis platform used in our study. Across 20 mice, the relative abundance of A. *muciniphila* on arrival averaged ~ 15%, slightly over 2-fold over that observed in our 6-month-old JAX cohort. This high representation of *A. muciniphila* in the fecal microbiota markedly reduced after two months (~ 2.3%) and across generations (~ 1–3% in F1 to F3)^35^. The combined results from our work and from this independent study suggest that C57BL/6J mice from The Jackson Laboratory are preferentially colonized by this taxon and that *A. muciniphila* abundance depends on the birth environment and standard diet type.

It is worth noting that both male and female C57BL/6J mice were used by Roman and coworkers while our experimental design only included male animals. The relative abundance of *A. muciniphila* ranged between ~ 1–47% compared to ~ 1–12% in our JAX cohort. It is possible that sex differences may exist, and further research will be needed to rigorously determine the role of sex on the relative abundance of *A. muciniphila* in mice.

Overall, these results from our study demonstrate for the first time the influence of SD and birth location on hippocampal-dependent spatial memory and gut microbiome in congenic C57BL/6J mice. Our findings are consistent with other studies using congenic C57BL/6J mice from The Jackson Laboratory in which *A. muciniphila* abundance is specifically enriched and we demonstrate that feeding mice with 5K52, the primary formula used by The Jackson Laboratory, maintains that enrichment for months. The impact of this work is particularly important for behavioral studies relying on hippocampal function because it readily indicates that C57BL/6J mice purchased from The Jackson Laboratory should not be immediately used alongside mice generated in a different institution. Accordingly, we recommend the mandatory inclusion of these factors in all neurobehavioral studies.

## Figures and Tables

**Table 1:** Table summary of the key differences between 5K52 and 2918 standard diets.

	2918 (Inotiv^®^)		5K52 (LabDiet^®^)		
Component	%	ppm	%	ppm	Δ (2918-5K52)
**Crude Protein**	**18.4**		**19.5**		**−1.1**
**Fat (ether extract)**	**6**		**6.2**		**−0.2**
**Carbohydrate**	**44.2**		*NA*		
**Crude Fiber**	**3.8**		**3.9**		**−0.1**
**Neutral Detergent Fiber**	**14.7**		**15.2**		**−0.5**
**Ash**	**5.5**		**6.3**		**−0.8**
**Calories from Protein**	**24**		**22.307**		**1.693**
**Calories from Fat**	**18**		**15.962**		**2.038**
**Calories from Carbohydrate**	**58**		**61.731**		**−3.731**
Zinc		70		81	**−11**
Manganese		100		160	**−60**
Copper		15		10	**5**
Iodine		6		2.2	**3.8**
Iron		200		360	**−160**
Vitamin A		15		20	**−5**
Vitamin D3		1.5		4.4	**−2.9**
Vitamin E		110		45	**65**
Vitamin K3 (menadione)		50		20	**30**
Vitamin B1 (thiamin)		17		79	**−62**
Vitamin B2 (riboflavin)		15		9.1	**5.9**
Niacin (nicotinic acid)		70		86	**−16**
Vitamin B6 (pyridoxine)		18		10	**8**
Folate		4		1.9	**2.1**
Choline		1200		1630	**−430**
Cholesterol		0		243	**−243**
Energy Density (kcal/g)	**3.1**		**3.14**		**−0.04**
Sterilization	*irridiated*		*non-irridiated*		

**Table 2:** Differential abundance of classified and unclassified species in the JAX cohort. The *P* values listed correspond to robust FDR *P* values.

Classified Species	Delta (%)	*P* value
*Alistipes* sp. UBA6068	−6.461	*0.0003*
*Akkermansia muciniphila*	−4.602	*0.0003*
*Enterorhabdus caecimuris*	−0.356	*0.0032*
bacterium 1XD42–54	0.122	*0.0007*
bacterium 1XD8–76	0.166	*0.0009*
bacterium 1XD21–70	0.253	*0.0022*
*Lachnospiraceae bacterium UBA7188*	0.331	*0.0014*
Unclassified Species	Delta (%)	P value
Akkermansia	−4.279	*0.0003*
Burkholderiales	−0.419	*0.0003*
Eubacterium	0.008	*0.001*

**Table 3.** Differential abundance of classified and unclassified species in the UMN cohort. The *P* values listed correspond to robust FDR *P* values.

Classified Species	Robust FDR PValue
*Bacteroidales* bacterium M1	*1.00E-105*
*Muribaculaceae* bacterium DSM 100749	*1.30E-79*
bacterium J10(2018)	*1.30E-24*
Bacteroidales bacterium M12	*6.10E-20*
*Prevotella* sp. MGM1	*2.60E-20*
*Bacteroidales* bacterium H6	*1.30E-13*
*Helicobacter* sp. MIT 03-1616	*1.70E-13*
*Lactobacillus intestinalis*	*6.80E-10*
*Lactobacillus* sp. ASF360	*7.40E-10*
*Porphyromonadaceae* bacterium UBA7154	*3.92E-09*
*Porphyromonadaceae* bacterium UBA7053	*6.41E-09*
*Enterorhabdus caecimuris*	*9.96E-09*
*Porphyromonadaceae* bacterium UBA7213	*1.11E-08*
*Bacteroidales* bacterium UBA714	*2.45E-08*
*Faecalibaculum rodentium*	*5.51E-08*
*Bacteroidales* bacterium M5	*3.66E-07*
*Lactobacillus reuteri*	*6.36E-07*
*prevotellaceae* bacterium UBA713	*1.55E-06*
*Helicobacter typhlonius*	*3.34E-06*
*Porphyromonadaceae* bacterium UBA3268	*7.61E-06*
*Alistipes timonensis*	*2.58E-05*
*Lactobacillus johnsonii*	*6.28E-05*
*Muribaculaceae* bacterium DSM 100764	*0.00025*
*Bacteroidales* bacterium M11	*0.0003*
*Bacteroidales* bacterium M14	*0.00039*
*Bacteroidales* Sp. UBA3406	*0.00058*
*Bacteroidales* bacterium M13	*0.00062*
*Desulfovibrio* sp.	*0.0009*
*Muribaculaceae* bacterium DSM 100746	*0.0013*
*Bacteroidales* bacterium M6	*0.00148*
*Muribaculaceae* bacterium DSM 103720	*0.00165*
*Bacteroidales* bacterium M10	*0.00335*
*Bacteroides acidifaciens*	*0.00337*
bacterium 1XD42-54	*0.00777*
Unclassified Species	Robust FDR PValue
Betaproteobacteria unclassified	*1.60E-43*
Proteobacteria unclassified	*1.60E-29*
Mesorhizobium unclassified	*9.50E-28*
saccharomyceta unclassified	*3.20E-19*
Prevotella unclassified	*4.20E-12*
Prevotellaceae unclassified	*2.70E-10*
Lactobacillus unclassified	*2.65E-08*
Akkermansia unclassified	*1.16E-06*
Porphyromonadaceae unclassified	*0.00188*
Muribaculaceae unclassified	*0.00705*

## Data Availability

All data needed to evaluate the conclusions in the paper are present in the paper and the Supplementary Materials. The data can be provided following scientific review and a completed material transfer agreement. Requests for data and materials should be submitted to the corresponding author.

## Abbreviations

BCM: Barnes circular maze
SD: standard diet
WD: Western diet
YM: Y maze
